# *Parechovirus*: neglected for too long?

**DOI:** 10.1128/jvi.01846-24

**Published:** 2025-03-25

**Authors:** Fahmida Alam, You Li, Matthew R. Vogt

**Affiliations:** 1 Department of Microbiology and Immunology, The University of North Carolina at Chapel Hill 318275 , Chapel Hill, North Carolina, USA; 2 Department of Pediatrics, Division of Infectious Diseases, The University of North Carolina at Chapel Hill 549964 , Chapel Hill, North Carolina, USA; Indiana University Bloomington, Bloomington, Indiana, USA

**Keywords:** picornavirus, parechovirus

## Abstract

Parechoviruses are non-enveloped, positive-sense, single-stranded RNA viruses that have been isolated from multiple vertebrate species. Infection with these etiologic agents of typically mild childhood respiratory and gastrointestinal illness in humans is nearly universal, and a subset of infected neonates and infants develop severe neurologic diseases. Rodent parechoviruses cause myocarditis, encephalitis, and perinatal death in multiple rodent species. The key steps of the viral life cycle, clinical characteristics, and global burden of these viruses are not well characterized yet, particularly for nonhuman parechoviruses. Here, we review the history of human and nonhuman parechovirus isolation, global seroprevalence and distribution, viral biology, and evolution, considering these factors might contribute to host specificity, virulence, tissue tropism, pathogenesis, host immunity, and population dynamics.

## INTRODUCTION

**Fig 1:**
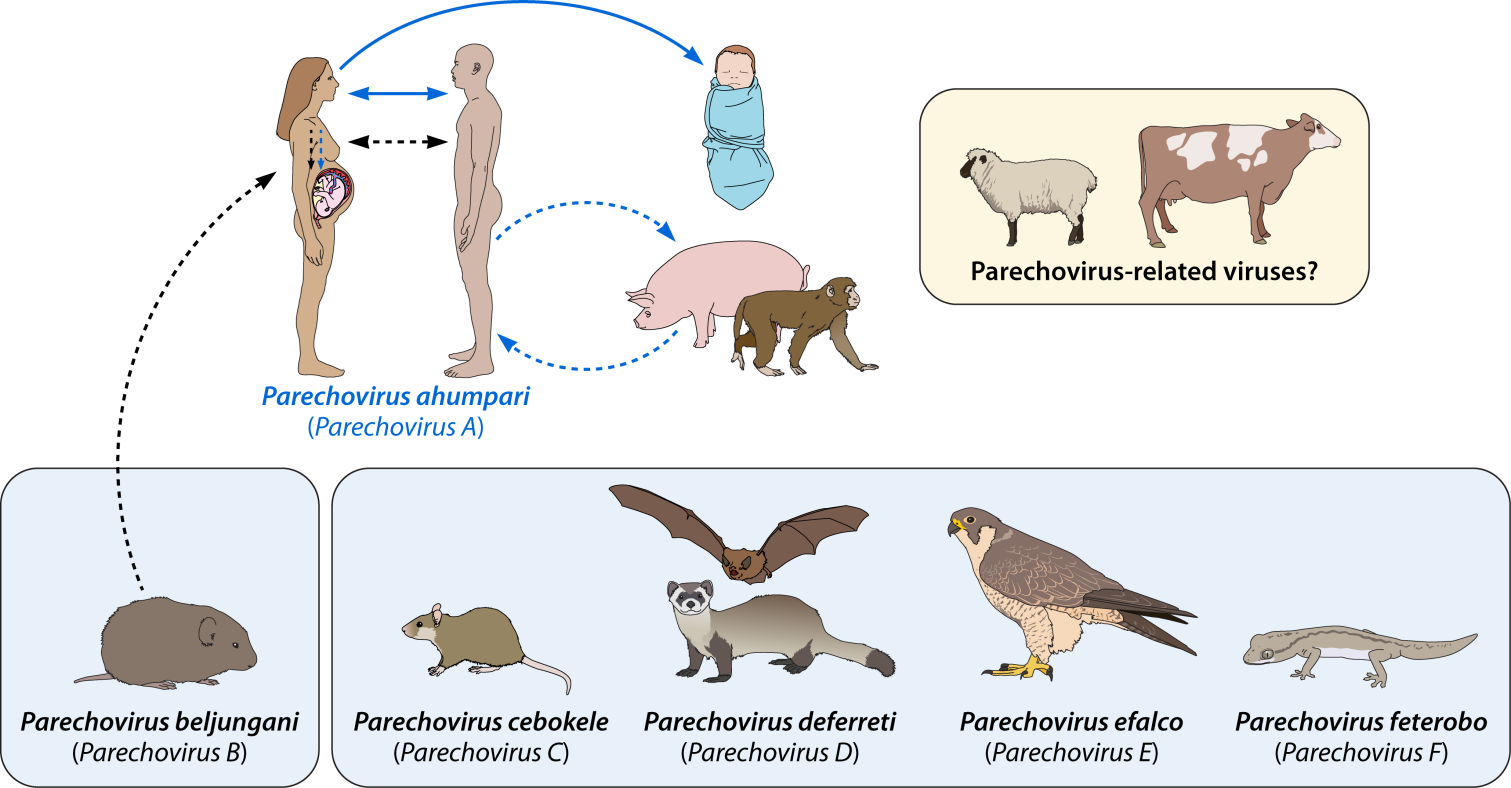
Host range of parechoviruses and directions of transmission. *Parechovirus A* genotypes were isolated from human specimens. *Parechovirus B*/Ljungan virus and *Parechovirus C* strains were isolated from rodents. *Parechovirus D* strains were isolated from ferrets and bats. *Parechovirus E* and *Parechovirus F* were isolated from falcons and geckos, respectively. Confirmed transmission directions are depicted with solid arrows, and potential transmission directions are represented with dashed arrows. Parechovirus-related picornaviruses that share nucleotide sequence and RNA secondary structure similarity with parechoviruses were isolated from asymptomatic ovines and bovines.

Parechoviruses belong to the large family of small RNA viruses called *Picornaviridae* (pico—small, RNA). Picornaviruses are clinically and economically important viruses characterized by a small icosahedral protein capsid structure that contains a single-stranded, positive-sense viral RNA genome (1). These viruses show great diversity in their host tropism, usage of viral proteins, exploitation of host lipid and protein functions at different steps of the viral life cycle, and disease manifestations in humans and other vertebrate and invertebrate hosts. The *Parechovirus* genus is one of the *Picornaviridae* family genera, which contains viruses within six parechovirus species, *Parechovirus A-F*, that infect multiple vertebrate species (Fig. 1). Recent parechovirus-associated pediatric disease outbreaks in different parts of the world (2–4) highlighted the importance of dedicated surveillance and a greater understanding of the biology and disease manifestations caused by these understudied viruses. In this review, we summarize the history of human and nonhuman parechovirus isolation, global seroprevalence and distribution, viral biology, and evolution, considering these factors might contribute to their host specificity, virulence, tissue tropism, pathogenesis, host immunity, and population dynamics.

## HISTORY OF PARECHOVIRUS ISOLATION

**Fig 2:**
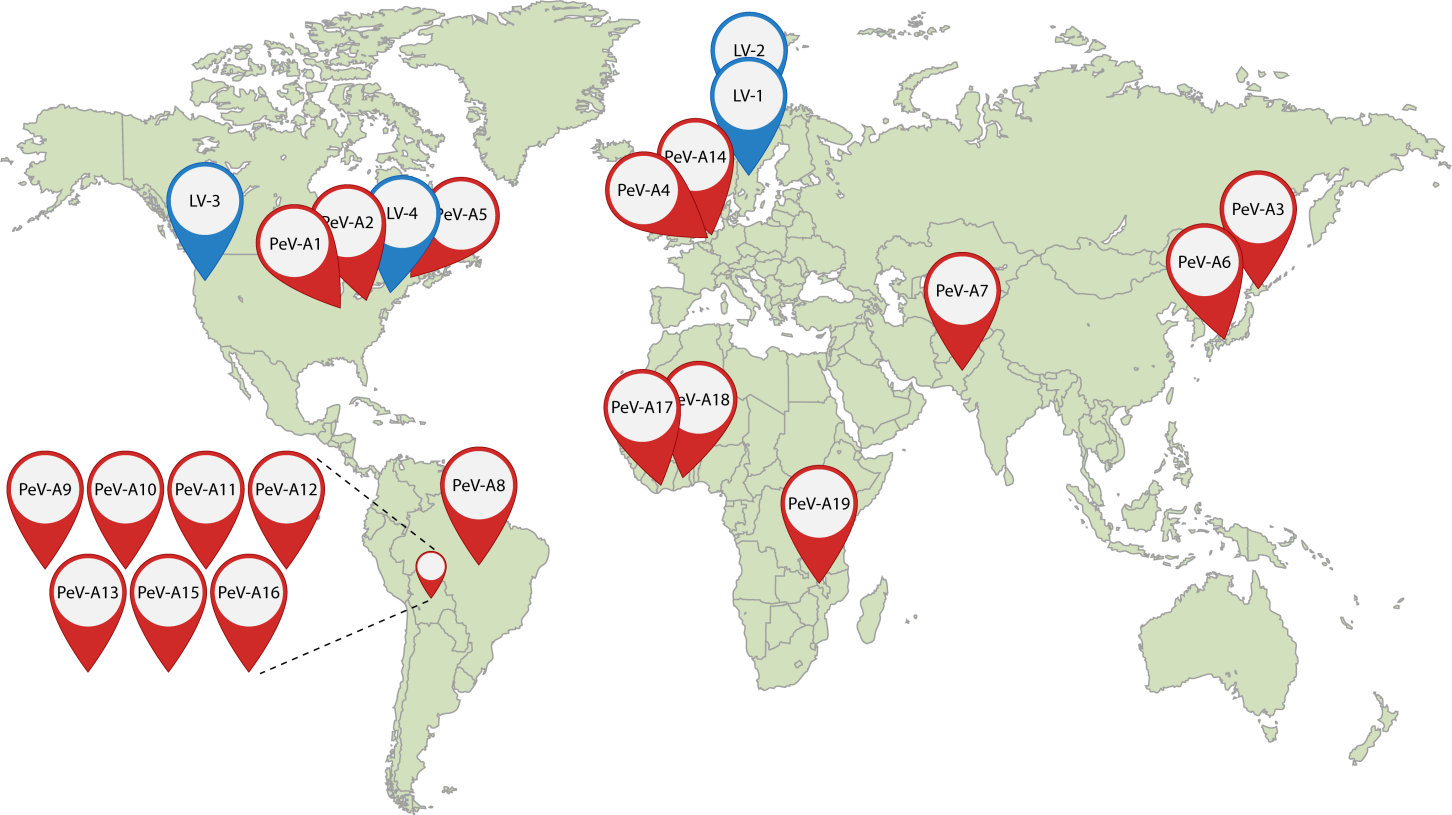
Geographic location of initial parechovirus isolation. The map depicts the locations where *Parechovirus-A* (PeV-A) (red) and *Parechovirus-B* (PeV-B) (blue) genotypes were initially isolated. PeV-A genotypes were initially isolated from the United States (PeV-A1, PeV-A2, PeV-A5); Japan (PeV-A3 and PeV-A6); The Netherlands (PeV-A4 and PeVA14); Pakistan (PeV-A7); Brazil (PeV-A8); Bolivia (PeV-A9, PeV-A10, PeV-A11, PeV-A12, PeV-A13, PeV-A15, PeV-A16); Ivory Coast (PeV-A17); Ghana (PeV-A18); and Malawi (PeVA19). PeV-B/LV genotypes were isolated from Sweden (LV-1 and LV-2) and the United States (LV-3 and LV-4).

As of 2025, the human-infecting parechovirus species *Parechovirus ahumpari/Parechovirus A* (PeV-A) has 19 total genotypes, designated as PeV-A1 through PeV-A19, isolated and typed from different parts of the world (Fig. 2). Initially isolated by Albert Sabin and Reinhard Wigand from pediatric fecal samples during the summer of 1956, the first two isolates of PeV-A were assigned *ECHO* (enteric, cytopathic, human, orphan) virus serotypes 22 and 23 (5). The classification of these later shifted to echovirus 22 (E22) and echovirus 23 (E23) within the *Enterovirus* (EV) genus. The complete genome sequencing of E22 and E23 in 1992 suggested that these viruses belong to an independent group of picornaviruses (6). Consequently, in 1997, the International Committee on Taxonomy of Viruses (ICTV) assigned these viruses a new genus, *Parechovirus*—Par (Greek *pará*)—close to ECHO. While earlier literature might abbreviate these viruses as HPeV or ParV, the current recommendation of the ICTV supports our abbreviation schema (7).

The original E22 and E23 isolates are now known as reference strains PeV-A1 (Harris) and PeV-A2 (Williamson), respectively (5). In 1986, an E23 virus was isolated from a child with a high fever in Connecticut and was later reclassified as a novel genotype in 2007, PeV-A5 (8). In 1999, an additional genotype of PeV-A, PeV-A3, was isolated from a 1-year-old child with transient paralysis and was reported in 2004 (9). Between 2004 and 2019, rapid genome sequencing enabled the identification of 16 additional PeV-A genotypes from distant parts of the world based on the sequence of the structural protein VP1. PeV-A4 was isolated from the fecal sample of a neonate with fever and poor feeding (10), PeV-A6 was isolated from cerebrospinal fluid (CSF) specimen of a 1-year-old child with Reye syndrome (11), and PeV-A8 was isolated from fecal samples of children with enteritis who are less than 6 years old (12). PeV-A9 through -A13, PeV-A15, and PeV-A16 genotypes were isolated from fecal samples of children with acute flaccid paralysis, although these viruses were not declared the causal agent of their neurologic symptoms at the time of isolation (13). PeV-A14 was isolated from fecal samples of children younger than 5 years (14). PeV-A17 was detected in a stool sample of a healthy 9-month-old child (15). PeV-A18 was isolated from pediatric fecal samples from children enrolled in a study investigating the etiologic agent of diarrhea (16). PeV-A19 was isolated from stool samples of children between 6 and 60 months of age with or without severe anemia (17).

*Parechovirus beljungani/Parechovirus B* (PeV-B), also known as *Ljungan virus* (LV), is associated with myocarditis, encephalitis, perinatal deaths (18), and diabetes (19) in multiple rodent species. PeV-Bs/LVs were assigned a separate *Parechovirus* species in 2005. Three LV isolates, the prototypical 87-012, 174F, and 145SL, were initially isolated from Swedish bank voles in 1999, the most abundant mammal in Sweden, when seeking an etiologic agent of lethal myocarditis in young athletes (20). LV 87-012 and 174F strains belong to the first genotype of PeV-B/LV, PeV-B1/LV-1, based on the nearly identical genome sequence, whereas 145SL belongs to the LV-2 genotype (21, 22). P1 structural region analysis of the fourth LV strain, M1146, isolated from Northwestern United States (Oregon), revealed a third genotype, LV-3, distinct from the Swedish LV strains (22). The fifth LV strain, 64-7855, was isolated from a red-backed vole in the Northeastern United States during an arbovirus study and belongs to a fourth genotype LV-4, based on VP1 and 3D region sequences (22).

Parechovirus C1 (PeV-C1) is a rodent virus of the *Parechovirus cebokele/*PeV-C species that was isolated in 1972 and characterized as an unclassified arbovirus until the complete genome sequencing and molecular analysis revealed it as a parechovirus, denoting a new species *Parechovirus C* (23). Parechovirus D1 (PeV-D1) or ferret parechovirus (FPeV, also referred to as MpPeV1 for the ferret species *Mustela putorius furo*) was isolated from rectal swabs of household ferrets, and based on amino acid identity and genome organization, it was assigned the first genotype of the fourth parechovirus species, *Parechovirus deferreti/PeV-D* (24). In 2019, a PeV variant, BtPeV QAPp32, was isolated and sequenced from the gut tissues of bats, which shared more than 70% nt and amino acid similarity with FPeV (25), and subsequent studies reported the identification of PeVs in bats (26, 27). Parechovirus E1/falcon parechovirus (FaPeV), detected in feces of birds of prey, is the only identified genotype of *Parechovirus efalco/*PeV-E species, and complete genome sequencing of Ljungan/Sebokele-like viruses revealed that they belong to a separate species (28). Gecko parechovirus (GPeV) is the only virus that belongs to the last species of PeV identified so far, *Parechovirus feterobo/*PeV-F (29). In summary, parechovirus has been isolated from multiple vertebrate animal species, and the early classification of PeV-A1 and PeV-A2 as EVs likely reduced the isolation of additional PeVs until readily available sequencing technologies increased the known spectrum of this genus.

## CLINICAL MANIFESTATIONS OF PARECHOVIRUS INFECTION

The disease severity experienced from PeV-A infection depends on the virus genotype and the infected individual’s age. Acute symptoms of PeV-A infection include, but are not limited to, fever, rash, nausea, vomiting, diarrhea, poor feeding, upper respiratory infection, weak muscle tone, seizures, sepsis-like illness, and central nervous system infection (30). Diseases caused by PeV-A infection range from asymptomatic to self-limited mild respiratory and gastrointestinal infection to severe neurologic diseases, including long-lasting neurologic sequelae with cerebral white matter damage, subcortical white matter cytotoxic cerebral edema, epilepsy, cerebral palsy, and learning disabilities in children (31–34). PeV-A infections were the second most common cause of viral sepsis-like illness and meningitis in neonates and infants in one study, following EVs (35). These viruses spread through the respiratory (36) and fecal-oral routes (30) in both asymptomatic and symptomatic patients. Most children experience asymptomatic to mild PeV-A infection by age five (37–39). PeV-A1, endemic in humans and the most frequently isolated genotype, can cause mild respiratory and gastrointestinal diseases. PeV-A3 can cause neurologic symptoms such as sepsis-like illness, acute flaccid paralysis, and meningoencephalitis (30), with most severe infections detected in infants younger than 3 months of age (30, 40). A 1.6%–6% mortality rate was reported only in infants with severe neurologic disease (34, 41). PeV-A3 infection is now recognized as an etiologic agent of acute neurologic diseases with long-lasting sequelae in infected neonates and infants (32, 33, 42, 43). While typically isolated from children, PeV-A infection is occasionally detected in immunocompromised adults (44, 45). Rodent-borne PeV-B/LV is associated with human diseases, including intrauterine fetal death and Type 1 diabetes in children, although causality is not established for either (46–48). Overall, PeVs can cause severe disease, especially in children, yet there are no US Food and Drug Administration-approved vaccines or antivirals targeting these viruses.

## GLOBAL SEROPREVALENCE AND DISTRIBUTION OF PARECHOVIRUS

PeV-A circulates globally, but the true prevalence of PeV-A is difficult to estimate as serologic surveillance data are limited. There is no global comprehensive active surveillance program for detecting, diagnosing, and typing PeV-A infection. Syndromic-based testing for PeVs is rare except for instances of severe neurologic symptoms. In the United States, the CDC maintains a passive, voluntary surveillance system known as the National Enterovirus Surveillance System (NESS) to track severe cases of EVs and PeV-As. In the last 6 years, NESS has reported a total of 94 cases: 2023, 4 (A3, A4, A6); 2022, 77 (A3, A4, A6); 2021, 3 (A1); 2020, no reports; 2019, 2 (A3); and 2018, 8 (A3, A6) (49). However, this is a gross underrepresentation of true PeV incidence in the United States that highlights the limits of passive surveillance, as a retrospective study over a nearly identical period (2017–2022) at a single medical center in Indiana identified 32 neuroinvasive PeV infections (50). There is no dedicated surveillance in Europe for parechovirus collection, and the Non-polio Enterovirus (NPEV) Surveillance System provides limited data on PeV-A (51). Clinical presentations of PeV-A infection tend to be indistinguishable from bacterial sepsis and meningitis, with inflammatory markers in the blood or CSF parameters (proteins, glucose, presence of inflammatory cells) often being normal (2, 32, 52), making it difficult to diagnose PeV infection based on clinical evaluation alone. Given the increasing awareness of the high relative risk of severe illness in infants with PeV-A3 infection, more active environmental assessment of parechovirus in human populations might be of value.

Neutralizing antibodies (nAbs) against PeV-A1 are nearly universal in adults (53–55). Anti-PeV-A3 nAb is present in ~70% of adults globally (56). In Finland, the Netherlands, and Japan, the seroprevalence of anti-PeV-A2 and -PeV-A4-6 nAbs is 60%–99% (53). The seroprevalence levels suggest that infection with one or more PeV-As by adulthood is a nearly universal experience. Lack of or low levels of maternal anti-PeV-A3 nAb can make neonates susceptible to serious diseases caused by the genotype (57). Worldwide PeV-A distribution differs by genotype. PeV-A1 is the most prevalent genotype in the United States, Asia, and Europe, followed by PeV-A3 and PeV-A4 (58). PeV-A6 is reported as the second most common in Australia (59) and in some studies from Europe (58). Although rare in Europe and the United States, PeV-A2 and A7–A19 have been reported in India, Pakistan, and Africa (58). While PeV-A infection often peaked in even-numbered years in the United States and certain European countries, Spain reported PeV-A detection peaks in late summer months in odd-numbered years (51). In a seroepidemiologic study in Finland, 36% of study participants had LV145SL-reactive antibodies, with a higher prevalence in urban areas, suggesting the possibility of human-to-human transmission of LV or antigenically related viruses (60). Altogether, these data indicate that evidence of PeV infection in humans is nearly universal by adulthood, with prevalence of subtypes varied by geography, but our ability to diagnose these infections acutely is still limited.

## PARECHOVIRUS BIOLOGY

**Fig 3:**
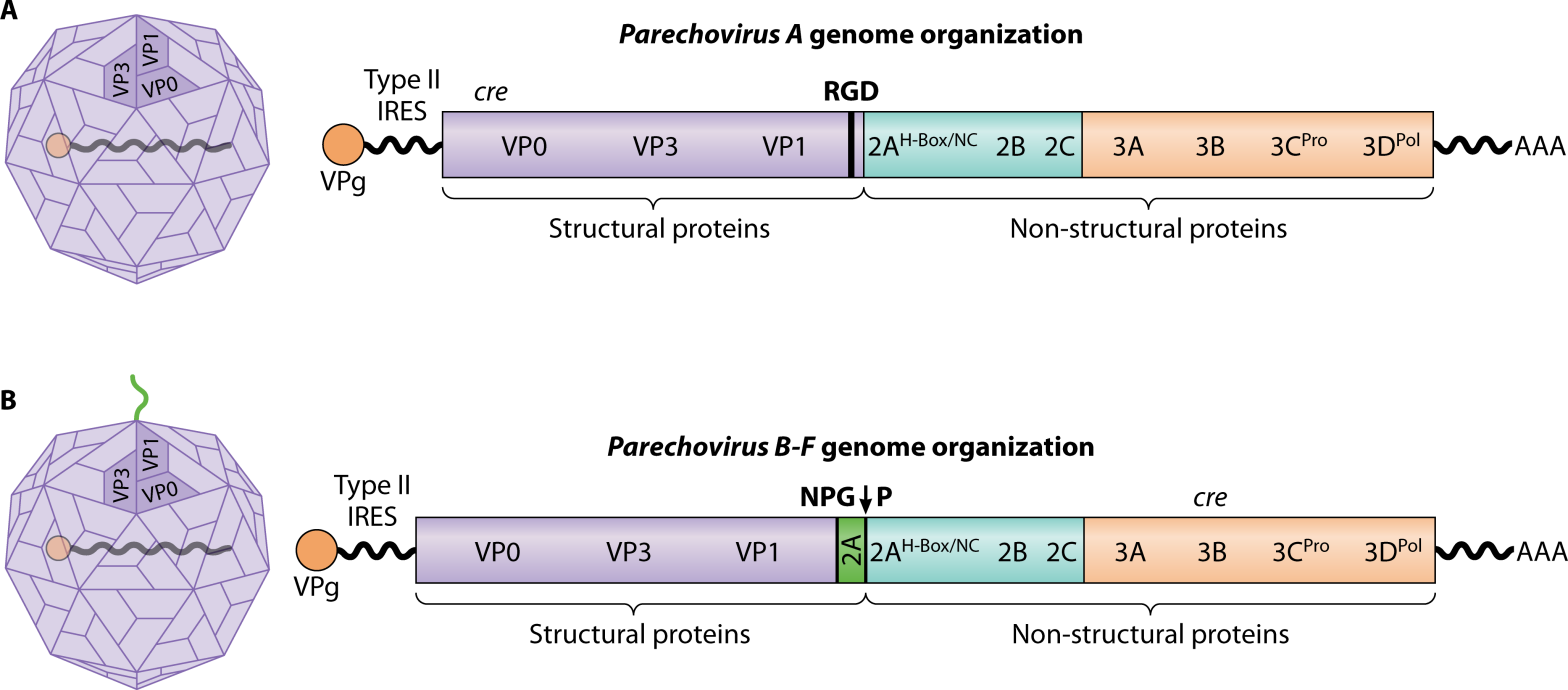
Parechovirus genome organization. (A) Viruses in the *Parechovirus A* species have cis-acting RNA elements (*cre*) in the structural protein-encoding region of the genome, contain only one 2A protein with an H-box/NC motif, and certain strains contain an arginine-glycine-aspartic acid (RGD) motif in the VP1 C-terminus. (B) Members of the *Parechovirus B-F* species have two separate 2A proteins: (i) a prototypical picornavirus 2A protein with an asparagine-proline-glycine-proline (NPG↓P) ribosome skip site sequence, and (ii) 2A2 protein containing H-box/NC motif. Parechovirus B genotypes presumably contain *cre* elements in the 3B region of the genome. *Cre* elements for other parechovirus species remain uncharacterized. Current evidence suggests that all parechoviruses contain a type II IRES.

PeVs share the overall structural and genomic organization of other picornaviruses but are distinct from other genera in many specific details (Fig. 3). Table 1 compares key characteristics of PeVs with other clinically significant genera in the *Picornaviridae* family with a more detailed description as follows:

**TABLE 1:** Comparison of key characteristics of clinically important *Picornavirus* genera^*a*^

	*Enterovirus*	*Parechovirus*	*Hepatovirus*	*Aphthovirus*	*Cardiovirus*	*Kobuvirus*	References
Genome length (nt)	7,100–7,450	7,339–7,608	7,487	7,250–8,203	7,730–8,530	Up to 8,200	(6, 61–70)
Structural proteins	VP4, VP2, VP3, VP1	VP0, VP3, VP1	VP4, VP2, VP3, VP1	VP4, VP2, VP3, VP1	VP4, VP2, VP3, VP1	VP0, VP3, VP1	(6, 63, 65, 66, 70, 71)
VP4 myristylation	Yes	No	No	Yes	Yes	Yes	(72–78)
Type of IRES	Type I	Type II	Type III	Type II	Type II	Type IV, V	(61, 79–85)
Poly (C)/polypyrimidine	Polypyrimidine tract	Polypyrimidine-like	Pyrimidine-rich	Poly (C)	Poly (C)	Pyrimidine tract	(66, 84, 86–90)
Protease(s)	2A^pro^, 3CD, 3C^pro^	3C^pro^	3C^pro^	L^pro^ and 3C^pro^	3C^pro^	3C^pro^	(91–93)
2A protein	Chymotrypsin-related endopeptidase	H-box/NC motif NPG↓P	pX protein	NPG↓P	NPG↓P	H-box/NC motif	(94–101)
L protein	No	No	No	Yes	Yes	Yes	(61, 66, 70, 102)
Pocket factor	Yes	No	No	No	No	No	(103, 104)

^*a*^ IRES, internal ribosome entry site; NPG↓P, asparagine-proline-glycine-proline; EMCV, encephalomyocarditis virus.

### Structure

PeVs are about 30 nm in diameter, spherical viruses with a smooth morphology. The naked PeV-A virions do not contain host-derived lipid envelopes. Virus-encoded protein capsids contain a positive-sense, single-stranded RNA genome. These viruses are acid stable (72), heat stable at 50°C (105), and the buoyant density in cesium chloride is 1.36 g/mL (106).

### 5′ and 3′ untranslated regions

PeVs have 7,300–7,600 nucleotide-long genomes. The only PeV-A open reading frame (ORF) is flanked by a 5′ untranslated region (UTR) and a 3′ UTR. The 5′ UTR of the genome forms a phosphodiester bond with the small genome-linked viral protein (VPg) using the aromatic side chain of the conserved third amino acid of the protein, tyrosine (107). The viral ORF is translated as a single polypeptide and then posttranslationally cleaved into mature protein precursors: P1, P2, and P3. The 5′ UTR of the PeV-A1 genome forms long hairpin structures, including stem-loops and a type II internal ribosome entry site (IRES) (86) by folding, which facilitates 5′ cap-independent translation initiation. The poly (C) tracts found in the 5′ UTR region of some picornaviruses, such as encephalomyocarditis (EMCV), are not present in PeV-A1 (86). There is an A/C-rich region between the two stem-loops and type II IRES within the 5′ UTR (86). PeV-A1 5′ UTR has a polypyrimidine tract about 20 nt upstream of the start codon, AUG, the same as observed in aphthoviruses, cardioviruses, and enteroviruses (86). The 3′ UTR of the genome is about 300 nt long and contains two highly conserved repeats of the sequence AUUAGAACACUAAUUUG arranged in tandem (8). Mfold analysis of the 3′ UTR suggested the composition of a single stem-loop structure with extensive base pairing between the U-rich 5′ end of the 3′ UTR and the poly (A) tail (8).

### Structural and non-structural proteins

Posttranslational processing of the P1 region of the PeV-A genome results in three structural proteins: VP0 (1AB protein, 38 kDa), VP3 (1C protein, 30 kDa), and VP1 (1D protein, 30.5 kDa). Notably, for other picornaviruses like EVs, the final maturation of VP0 protein requires viral genome-induced cleavage of the protein into VP4 and VP2 proteins. However, the PeV VP0 is not cleaved (6), so they lack the small VP4 protein inside the capsid, and the corresponding VP4-like N-terminus of VP0 is not myristoylated (72). A structural study suggested that the N-terminus of the VP0 protein stabilizes the inner surface of the capsid (108). Sixty copies of each capsid protein form an icosahedral capsid with pseudo-triangulation number (T)=3 symmetry, resulting in fivefold, threefold, and twofold axes of symmetry. Each of the capsid proteins forms an eight-stranded antiparallel β-barrel, strands B through I, forming wedge-like structures (109). Unlike many picornaviruses, the PeV-A capsid surface is relatively flat and lacks the classic VP1 hydrophobic pocket. Shorter surface exposed loops create a shallow depression instead of a canyon around the fivefold axis of symmetry (109). As a result, PeVs do not have fatty-acid pocket factors found in other picornaviruses for stabilizing the hydrophobic pocket. The shallower depressions on PeVs are occupied by bulky amino acid side chains in PeV-A (108) and LV (94), rendering small molecule capsid-binding inhibitors ineffective (110). The relatively longer C-terminus of LV VP1 creates distinct protrusions on the capsid surface around the fivefold axis of symmetry (94). There is a *cis*-acting replication element (*cre*) in the 1AB/VP0 region (the β G strand) of the PeV-A genome and the 3B region of the LV genome (8). A conserved CAAAC motif, as in multiple other picornaviruses, is present within the predicted stem-loop structure of PeV-A and LV *cre* (8). PeV-A1 and PeV-A2 VP3 regions have 30 amino acid-long, highly basic N-terminal loops, not observed in other picornaviruses (79).

The P2 and P3 regions are cleaved into seven nonstructural proteins, 2A-C and 3A-D, that play important roles in the viral life cycle. PeV-A 2A contains a conserved H-box/NC motif and a putative transmembrane domain that is characteristic of a family of cellular proteins involved in cell proliferation and phospholipid metabolization called H-rev107 or phospholipase A and acyltransferase 3 (PLA/AT3) (95). Interestingly, PLA/AT3, also known as PLA2G16, is a pan-enterovirus host factor that enables viral genome delivery into the cytoplasm (111, 112). PeV-B-F species have two unrelated 2A proteins in their genome, an asparagine-proline-glycine-proline (NPG↓P)/2A1 protein and a histidine, asparagine, and cysteine dipeptide (H-NC box)/2A2 protein. LV encodes an aphthovirus-like 2A protein with a DvExNPGP core sequence motif (113). Polyprotein processing by the NPG↓P ribosome skip site results in a unique 42-43 amino acid extension at the C-terminus of LV VP1 protein (94). 2A protein has been shown to preferentially bind to the 3′ UTR of single-stranded viral RNA and has an affinity for the 3′ UTR of viral dsRNA, suggesting a role in viral replication (114). 2B is a small hydrophobic protein, which, if expressed individually in cells, co-localizes in the endoplasmic reticulum (ER) with ER marker B cell receptor-associated protein 31 (BAP31) (115). PeV-A1 2C is predicted to be a helicase with magnesium-dependent ATP diphosphohydrolase activity (116). 3B encodes the previously described VPg protein. 3C and 3D encode the only protease and the RNA-dependent RNA polymerase (RdRp), respectively. The genome layout of PeV-A is VPg+5′-UTR^IRES-II^-[1AB-1C-1D-/2A^H-box/NC^-2B-2C/3A-3B^VPg^-3C^Pro^-3D^RdRp^]-3′-UTR-poly(A) (Fig. 3A). The genome organization of PeV-B-F is VPg+5′-UTR^IRES-II^-[1AB-1C-1D-/2A^NPG↓P^-2A^H-box/NC^-2B-2C/3A-3B^VPg^-3C^Pro^-3D^RdRp^]-3′-UTR-poly(A) (Fig. 3B).

### Virus entry

PeV-As that contain the integrin-binding tripeptide arginine-glycine-aspartic acid (RGD) motif at the C-terminus of the VP1 protein utilize integrins (α_5_β_1,_ α_5_β_3_, α_5_β_6_) on host cell surfaces (117, 118) as attachment factors for viral entry. Heparan sulfate and beta-2-microglobulin play a role in PeV-A virus binding to certain cells (58). Human myeloid-associated differentiation marker (MYADM) also functions as an entry factor for PeV-A1, PeV-A2, and PeV-A3 (119, 120) and possibly for other PeVs whose entry mechanisms have not yet been well characterized. VP0 protein of PeV-A3 interacts with the fourth extracellular loop of MYADM for viral attachment/entry (119), and this MYADM loop is a critical determinant of species tropism for PeV-A1 (120). PeV-A3 strains generally lack an RGD motif on the VP1 C-terminus. Viral isolates from other genotypes lacking the RGD motif were reported, such as PeV-A1 (14), PeV-A5 (14), PeV-A10 (38), PeV-A11 (121), PeV-A13 (38), PeV-A15 (38), suggesting that it is not a unique characteristic of the PeV-A3 genotype. Isolates from other PeV species such as LV, PeV-C1, and PeV-D1 lack RGD motifs in their VP1 region. The viral attachment and uncoating receptors for PeV-A4-19 and PeV-B-F species have not been identified yet (94).

Upon binding to attachment/entry factors, PeV-A1 is endocytosed by a clathrin-dependent endocytic pathway (55). In PeV-A1-infected A549 cells, PeV-A1 co-localized with early endosomes (EEA1) and late endosomes (mannose-6-phosphate receptor) but not with caveolin-1 (55). In EVs, myristoyl-VP4 forms hexameric membrane pores that facilitate viral RNA release into the host cell cytoplasm (122). A structural study suggested that the N terminus of the PeV VP0 that stabilizes the inner surface of the capsid forms membrane pores (108). Given that PeV-As have no VP4 proteins and the protein is not myristoylated, the uncoating mechanism(s) of these viruses is likely to be different from EVs, but the mechanism is not known yet.

### Viral protein translation and RNA replication

We have limited experimental evidence of PeV protein translation and genome replication. Host ribosomes translate the positive-sense viral RNA into a polyprotein precursor. PeV-A1 infection in HeLa cells does not shut off host protein production because the virus does not cleave cap-binding protein p220, also known as eIF4G, which is essential for cap-dependent protein translation (123, 124). In this regard, PeV-A1 resembles EMCV of the *Cardiovirus* genus more than viruses in the *Enterovirus* genus, such as polioviruses (PVs), for which p220 cleavage is mediated by the 2A protease. During EMCV infection, instead of a direct shutoff of host protein production by viral protease activity, cap- and IRES-dependent translations compete for host translation machinery, and the virus outcompetes the host cells (87). Because PeV 2A lacks protease activity (125), 3C is the only viral-encoded protease involved in polyprotein processing. In a cell-free expression system, the recombinant PeV-A1 3C could cleave a VP1-2A precursor in *trans* but not the P2-P3 junction, suggesting either a requirement for an intramolecular 3C-mediated *cis* cleavage or a functional intermediate such as 3CD (125), although such intermediates have not been described for PeVs. For comparison, PV intermediate 3CD can proteolytically cleave the P1 capsid precursor to structural proteins (126, 127) despite lacking polymerase activity (91).

Like other positive-sense RNA viruses, the viral RNA of PeV-A is transcribed into (–)-sense RNA that serves as a template for more (+)-sense RNA formation. The replication complex (RC) of PeV-A1 induces subtle membrane alterations compared to EVs, as its replication does not require the host cell’s entire endomembrane system (128). By labeling nascent viral RNA with bromodeoxyuridine, it was shown that the replication complex of PeV-A1 consisted of dilated ER and a disintegrated Golgi that contained a trans-Golgi marker, 1,4-galactosyltransferase, and nonstructural protein 2C. The covalently linked VPg protein when uridylylated (VPg-pUpU) functions as a protein primer for RNA synthesis, like other picornaviruses (129). While brefeldin A (BFA) strongly inhibits PV RNA replication by blocking coat protein 1 (COPI)-mediated ER to Golgi transport, BFA only partially blocks PeV-A1 RNA replication, suggesting that even though PeV-A1 RCs are generated from COPI-associated membranes, COPI might not be directly involved in RC formation (130). The 2C proteins present in the RC lack ATPase activity, whereas ER-derived membrane-bound 2C, which lacks viral RNA, is active as an ATPase (128). PeV-A1 3A protein showed binding affinity to the Golgi adaptor acyl-coenzyme A (Acyl Co-A) binding domain protein 3 (ACBD3/GCP60), a factor picornaviruses use to recruit essential kinases like PI4KB to replication complex (131). Individually expressed 3A proteins of PeV-A1 localized with the Golgi without changing intracellular morphology (115). The 3D-encoding region has conserved domains that suggest RNA secondary structures may be important for PeV-A genome replication (132).

### Virion assembly and egress

PeV-A RNA contains packaging signals that guide the assembly of capsid proteins. Up to 60 unique interactions between stem-loop structures of viral RNA and conserved amino acids in the capsid proteins VP3 and VP1 have been identified (133, 134), and these interactions are credited to contribute to viral capsid packaging. Although empty capsids are characteristic of picornavirus replication, there has been a report of no observed procapsids when culturing PeV-A1 and PeV-A3 (108). PeV-As are assumed to be released from infected cells via cell lysis as cytopathic effects were observed, but non-cytopathic PeV-A3 infection in different cell lines was reported (135). Poliovirus (PV), coxsackievirus B3, and hepatitis A virus are among the picornaviruses that egress infected cells packaged into vesicles or envelopes hijacked from cellular membranes (136–138). It is plausible that PeV-A also uses non-cytolytic pathways to exit the cells, although, to our knowledge, there have been no reports of non-lytic pathways yet.

## MODELING PARECHOVIRUS INFECTION

A few studies have reported using organoid and small animal models to understand PeV-A1 and PeV-A3 tropism, pathogenesis, and host immune response.

### Organoid models

A two-dimensional fetal enteroid model has been used to study PeV-A tropism and replication using laboratory-adapted virus strains and clinical isolates. Basolateral inoculation promoted the highest rate of viral replication and apical shedding for both PeV-A1 and PeV-A3 (139). PeV-A1 infected Paneth cells and enterocytes, whereas PeV-A3 predominantly infected goblet cells (139). PeV-A infection was characterized in an unguided neuronal organoid model generated from human pluripotent stem cells. These organoids mature gradually, showing genetic characteristics of human embryonic development and containing neuronal cell types such as neurons and astrocytes; however, they lack microglia, a key immune cell type of the nervous system (140). PeV-A1 and PeV-A3 infections did not cause any significant changes to the architecture of these organoids, but both co-localized with astrocytes and neuron-rich areas within the organoid (140). Clinical isolates of PeV-A3 infection resulted in higher interferon-stimulated gene expression, but PeV-A1 did not induce the same inflammatory response (140).

### Small animal models

Murine T cells that were isolated from PeV-A1 intraperitoneal-inoculated C3H/HeJ mice recognized and proliferated in response to several enteroviruses such as coxsackieviruses A16, B2, B3, B6, and poliovirus 1 but not when stimulated with other picornaviruses like Mengo virus and Theiler’s murine encephalomyelitis virus of the *Cardiovirus* genus (123). This suggests that PeV-A1 might share T cell epitopes with certain enteroviruses but not universally with picornaviruses. A mouse model of neuroinvasive PeV-A infection was generated by inoculating 3-day-old wild-type C57BL/6 mice intracranially with 5 × 10^5^ PFU/mouse with PeV-A3 virus (141). The authors observed morbidity, mortality, and hindlimb paralysis in infected mice. PeV-A3 VP0 protein was detected in the hippocampus and cortex of the infected mice, suggesting these viruses are both neurotropic and neurovirulent in neonatal wild-type C57BL/6 mice. Additionally, brain tissues from infected mice had higher expression of inflammatory cytokines (interferon-γ, IL-6, IL-1-β, and TNF-α) and death signaling molecules (microtubule-associated protein 1A/1B-light chain 3 [LC3]-phosphatidylethanolamine conjugate [LC3-II], cleaved caspase 3, phosphorylated Nuclear factor kappa-light-chain-enhancer [NF-κB] p65, and cleaved Receptor-interacting serine/threonine-protein kinase 1 [RIPK1]). These models do not fully recapitulate natural PeV infection, though, as these viruses transmit via the fecal-oral and respiratory routes. The mechanism(s) by which these viruses, especially PeV-A3, cause neurologic diseases remains unknown. In the CD-1 mouse model of LV infection, pregnant female mice infected with LV typically had 90% of pups in their litters die perinatally. Furthermore, the surviving female pups that then reproduced up to 6 months later lost about 25% of their own pups compared to essentially zero loss in matched controls, suggesting either long-term viral persistence or altered host immunity in mice exposed to LV *in utero* (18). Developing and further studying animal models that better mimic the clinical features of PeV infection and disease are needed to enhance our understanding of PeV viral and immune pathogenesis.

## PARECHOVIRUS EVOLUTION AND GENETIC DIVERSITY

Genealogy-based coalescent approaches can estimate timed viral ancestry and the rates of genetic change using nucleotide sequences sampled over an epidemiological time frame (142, 143). Bayesian analysis comparing PeV-A1 through PeV-A8 showed that the entire structural protein-coding region P1 and the VP1 protein specifically have high substitution rates, 2.21 × 10^−3^ (0.48–4.21 × 10^−3^) and 2.79 × 10^−3^ (2.05–3.66 × 10^−3^) substitutions per site per year, respectively (144). Current estimation suggests that the circulating common PeV-A lineages diverged from their most recent common ancestor at around 1600 (1427–1733), about 400 years before their initial report of isolation and characterization as picornaviruses (144). Based on the VP1 sequence, it was postulated that PeV-A8 diverged from the same group as PeV-A3, PeV-A7, and PeV-A14 approximately 315 years ago; PeV-A14 diverged from PeV-A3 and PeV-A7 around 220 years ago; and PeV-A7 diverged from PeV-A3 around 150 years ago (144). In 2009, based on a 606 nt VP1 sequence comparison of 61 PeV-A isolates, the authors proposed that PeV-A1 viruses diverged into two distinct clusters termed clades 1A and 1B (132). In a more recent study using near full-length sequences, similar clustering of locally isolated PeV-A1 strains into 1A and 1B was observed (145). The authors concluded that the PeV-A1A and PeV-A1B clades diverged from each other at least 100 years ago and were not a direct result of local recombination (145). 3D^RdRp^ sequence-based phylogenetic comparison of variants collected from four geographically separated locations showed that PeV-A1B and PeV-A3 were the most frequently detected genotypes, with high differences in PeV-A1B, A5, and A6 recombinant form (RF) frequencies (146). Based on divergence in the VP3/VP1 region, PeV-A1 and PeV-A3 RFs have a half-life of 4 years and 20 years, respectively (146). The lower level of genomic diversity observed within PeV-A3 isolates might suggest a more recent emergence of this genotype and/or limited opportunity to recombine with other genotypes within a co-infected host cell because of biological and epidemiological constraints (147).

A ratio of synonymous (dS) and non-synonymous (dN) substitution of >1 is an indicator of positive selection at the amino acid level (148), whereas values significantly lower than 1 indicate purifying selection and preservation of the phenotypic trait. The frequency of dS and dN substitutions and the dN/dS ratio within the P1 region and the VP1 protein of PeV-A genomes indicate that purifying selection is the dominant evolutionary force, resulting in amino acid conservation (144). PeV-As showed amino acid divergence in the capsid-encoding regions and an elevated dN/dS ratio in the structural gene region compared to other regions of the genome (149), similar to EVs and foot-and-mouth disease virus.

Like other picornaviruses, intratypic and intertypic recombinations play critical roles in PeV diversity and evolution. The first report of PeV-A intertypic recombination was published in 2007, in which the authors identified a potential recombination between the structural genes of a PeV-A4 isolate and the non-structural regions of PeV-A3 (8). A novel recombinant PeV-A3 strain from a 2015 outbreak in Australia had a structural protein similarity of 99.6% to the Yamagata 2011 lineage, but non-structural proteins had less than 85% similarity to any known PeV-A isolated and sequenced thus far (3). The sequences downstream of the recombination breakpoint between VP1 and 2A could have resulted from single or multiple recombination events between PeV-A1, other lineages of PeV-A3, PeV-A4, PeV-A5, PeV-A6, or from unidentified animal reservoirs (3). PeV-A4 isolated from infants with sepsis-like illnesses in Finland had capsid-coding regions with high sequence similarity with other PeV-A4s, but non-structural protein encoding 2A-3C regions shared a sequence similarity with several PeV-A1, PeV-A3, and a 2002 PeV-A4 isolate from the Netherlands (K251176-02) but not with other PeV-A4s (150). The RdRp-encoding sequences of these viruses also lack similarities with the RdRp of K251176-02 (PeV-A4), forming unique phylogenetic clusters with PeV-A1 and PeV-A3 (150). Mosaic recombinant strains isolated from distant cities in rural Brazil showed unique recombination patterns throughout the genome except the VP1 region, with recombination observed between PeV-A1 and PeV-A5 (Rec1/5); PeV-A4, A5, and A17 (Rec 4/17/5); PeV-A1 and PeV-A17 (Rec1/17); and PeV-A1 and an unidentified strain (Rec1/und) (145). A potential recombination breakpoint between regions P2 and P3 was reported in a virus isolated from a child with diarrhea in China (151). The P1 and P2 regions of the CH-ZXY1 isolate phylogenetically clustered with PeV-A5 strains, and the P3 region clustered with PeV-A1 strains (151), with analysis showing recombination between the Brazilian PeV-A5 strain Br/53/2006 (HQ696575) isolated from fecal samples of patients with acute diarrhea (152) and the PeV-A3 strain BNI-788st (EF051629) isolated from stool samples of a patient in Hamburg, Germany (153). PeV-A3 is more conserved throughout the genome compared to PeV-A1, A4, A5, and A6, as recombination events for the latter genotypes were observed more frequently with recombination sites observed in regions P1 and P2 (147, 154). The northeastern LV4 64-7855 strain shared phylogenetic relationships with the Swedish LV2 145SL strain based on the central region of the genome and with the northwestern LV3 M1146 strain based on the rest of the viral genome, indicating that ancestral recombination might have resulted in the fourth genotype of the virus species (22). Because different PeV types are differentially virulent and can clearly recombine, just as isolates of live attenuated poliovirus vaccine strains can recombine with other *Enterovirus C* species isolates, understanding the molecular drivers of PeV recombination is important from a public health perspective.

## POTENTIAL FOR PARECHOVIRUS ZOONOSES

Parechovirus zoonosis is poorly characterized. However, human PeV-A strains were detected in domestic animals, farm animals, and synanthropic nonhuman primates (NHP) (13, 155). PeV-A4 detected in human and domestic porcine fecal specimens in rural Bolivia shared a 97%–100% nucleotide identity, suggesting cross-species transmission in the local community (13). PeV-A1, A4, A5, A12, A14, A15, and LV-related viruses were detected in fecal samples of synanthropic NHPs in urban Bangladesh (155). PeV-A1 and PeV-A6 were detected in monkeys with diarrhea in China (156). To our knowledge, PeV species that are endemic to simian and porcine species have not been identified/reported. Additionally, there is precedent for human picornaviruses recombining in nonhuman hosts. For example, two human enteroviruses, coxsackievirus A9 and B5, co-infected swine, and subsequent recombination gave rise to swine vesicular disease virus around 1960-1961 (157). Two closely related picornaviruses isolated from asymptomatic farm animals in Hungary, bovine hungarovirus 1 (BhuV-1) and ovine hungarovirus (OHuV-1), contained type II IRES, shared nucleotide sequence, and RNA secondary structure similarity with LV and PeV-A, particularly PeV-A3 (158). The extent of animal reservoirs for PeV species and the direction of existing and potential viral transmission are not well understood yet, which makes parechovirus zoonosis an interesting and consequential new area of study.

## PERSPECTIVES

Seven decades after their initial isolation, many key properties of human and nonhuman PeV biology, clinical characteristics, host range, and host immune response remain unknown. Recent PeV-A3 epidemics associated with neonatal encephalitis in different parts of the world (2, 42) garnered a renewed interest in these viruses. It is too early to determine whether PeV-A3 is a re-emerging virus or if increased awareness revealed the true, higher-than-previously-appreciated prevalence of the genotype.

While PeV-A is considered a self-limiting virus in humans, LV1 infection in female mice causes persistent pregnancy-related complications (18). The possibility of intrauterine infections has been reported in humans (159, 160), but vertical transmission of PeV-A and LV is not verified yet. PeVs are presumably horizontally transmitted from the mother or others shortly after birth. The lack of an animal model that recapitulates natural, systemic infection is a barrier to understanding how these viruses that spread by the respiratory and the fecal-oral routes cause neurologic disease, potential vertical transmission, viral persistence in tissues, and if direct infection or robust systemic inflammation is responsible for the broad range of disease manifestations observed.

Many key aspects of the PeV life cycle remain understudied, such as receptors and uncoating cues, generation of replication organelles, cellular proteins involved in replication, virion assembly, and maturation steps. Stable and functional intermediates that play key roles in EV life cycle, such as PV 3CD (161), have not been identified for PeV yet. Whether the PeV 2A protein, which is a homolog of cellular PLA/AT3—the cellular form being essential for EV entry—determines species specificity and confers any evolutionary advantage to PeVs remains unknown. Clearly, PeVs are not simply “other enteroviruses” and demand further research given their increasing recognition as a causative agent of severe neurologic disease.

PeV-A was isolated from domestic animals, but we are unaware of any known animal reservoirs of human parechovirus and the direction of virus transmission between humans, domestic animals, synanthropic nonhuman primates, and other species infected with PeVs. Our understanding of PeV genetic diversity and evolution and their impact on virulence, pathogenesis, population dynamics, and clinical significance is evolving, as more clinical, epidemiologic, and basic virology data become available. A comprehensive understanding of PeV prevalence through dedicated surveillance and the understanding of biology and pathogenesis will help us gauge the magnitude of the threat posed by these viruses, determine the best correlates of protection against PeV-induced severe neurologic disease, and prevent potential zoonotic outbreaks.
